# Fibrinogen and antithrombin III are associated with in-hospital mortality among critically ill patients with acute kidney injury

**DOI:** 10.1080/0886022X.2022.2142138

**Published:** 2022-11-10

**Authors:** Guangyuan Zhang, Lei Zhang, Sun Si, Tiancheng Jiang, Yi Xia, Yongkun Zhu, Xiangyu Zhang, Chi Yao, Ming Chen, Shuqiu Chen

**Affiliations:** a Department of Urology, Zhongda Hospital, Southeast University, Nanjing, China; b Institute of Urology, Medical School, Southeast University, Nanjing, China

**Keywords:** Coagulation parameters, fibrinogen, antithrombin III, acute kidney injury, in-hospital mortality

## Abstract

**Objectives:**

Coagulation factors participates in the inflammatory cascade, known to play a crucial role in the development of acute kidney injury (AKI). Thus, it’s likely that some factors may be associated with AKI. Among them, low levels of fibrinogen and antithrombin III (ATIII) activity have been proved to increase mortality in patients with sepsis. Moreover, they are also reported to be associated with higher incidence of AKI. However, the association between coagulation parameters, especially fibrinogen and ATIII, and prognosis of AKI has not been examined.

**Methods:**

Data were acquired from Multiparameter Intelligent Monitoring in Intensive Care Database IV (MIMIC-IV) version 1.0. Cox proportional hazards regression model was used to estimate the relationship between coagulation parameters and in-hospital mortality in critically ill patients with AKI. Subgroup analysis was also conducted to assess the robustness of the association. Restricted cubic spline (RCS) curve was utilized to examine the nonlinear relationships between fibrinogen or ATIII and in-hospital mortality. Kaplan–Meier method was used to estimate cumulative incidence of mortality by fibrinogen or ATIII levels. Receiver-operating characteristic (ROC) curve was plotted and area under curve was calculated to evaluate predictive ability of fibrinogen or ATIII.

**Results:**

A total of 5914 eligible patients were enrolled in fibrinogen cohort study and 115 patients were eligible for ATIII cohort study. The baseline of low fibrinogen (<150 mg/dL) or ATIII (<80%) activity was associated with significantly higher in-hospital mortality (fibrinogen HR [95% CIs] 2.01 [1.79, 2.27]; ATIII 3.73 [1.11, 12.54]). The HR [95% CIs] of low fibrinogen remained significant 1.29 (1.13, 1.48) in multivariate analysis. The RCS curve showed nearly linear relationship. Subgroup analysis also proved the robustness of the association between fibrinogen and in-hospital mortality. Kaplan–Meier survival curve and ROC demonstrated the predictive capability of fibrinogen and ATIII.

**Conclusion:**

Low fibrinogen is an independent predictor of in-hospital mortality in critically ill patients with AKI. Low ATIII activity is also likely to impact the risk of in-hospital death.

## Introduction

Acute kidney injury (AKI) is a severe clinical syndrome with high morbidity and mortality [1–3]. It is reported to be a common comorbidity in critically ill patients [4], which will increase length of stay (LOS), costs, risk of in-hospital death and long-term risk of death [5–7]. Clinical and preclinical researches suggested that the early detection and management of risk factors for AKI could decrease in-hospital mortality [8,9].

Inflammatory response gets involved in AKI, under circumstances of ischemic reperfusion and sepsis [10–12]. Inflammation was proved to interact with coagulation in many situations and some coagulation factors play a crucial role in inflammation response [13,14]. Specifically, the decreased levels of fibrinogen and antithrombin activity have been reported to be significantly associated with an increased mortality in patients with sepsis [15,16]. Theoretically, coagulation factors, like fibrinogen and antithrombin III (ATIII), are possibly related to AKI.

Fibrinogen is a serum glycoprotein that gets involved in coagulation and inflammation response, like regulation of macrophage adhesion [17,18]. A recent study reported that low fibrinogen was a risk factor for the development of AKI after liver transplantation [19]. Moreover, basic research indicated that exogenous fibrinogen-derived peptides administration protects mice from ischemia/reperfusion (I/R) kidney injury [20]. Similar to fibrinogen, clinical data revealed the vulnerability to AKI in patients with low ATIII activity, who underwent cardiac surgery [21], coronary arteriography [22], liver transplantation [23], or cesarean section [24].

In summary, some coagulation factors, specifically fibrinogen and ATIII, are related to AKI. However, the association between them and prognosis of AKI patients remains unknown. Here, we conducted the research to explore the prognostic value of fibrinogen and ATIII for critically ill patients with AKI.

## Materials and methods

### Data source

This retrospective study was based on Multiparameter Intelligent Monitoring in Intensive Care Database IV (MIMIC-IV) version 1.0 which is freely accessible to researchers. The database was an update to MIMIC-III and contained more than 60,000 ICU stays between 2008 and 2019, which was maintained by Beth Israel Deaconess Medical Center [25]. The Institutional Review Boards of the Massachusetts Institute of Technology (Cambridge, MA, USA) and the Beth Israel Deaconess Medical Center approved our access to the database after completing the National Institutes of Health’s web-based course, called Protecting Human Research Participants (certification number: 46538344).

### Population selection

Patients were diagnosed with AKI according to the Kidney Disease: Improving Global Outcomes (KDIGO) criteria. Specifically, an increase in serum criteria ≥ 0.3 mg/dL within 48 h or ≥1.5 times baseline or urine output <0.5 mL/kg/hour for 6 h. AKI stages were also defined by both serum creatinine (Scr) and urine volume during the first 48 h after ICU admission. Patients were excluded according to the following criteria: (1) no coagulation parameters measured during first 24 h after ICU admission; (2) age <18 years; (3) died within 2 days after ICU admission; (4) no complete clinical or laboratory data records; (5) re-admission. The overall in-hospital mortality is 11.7% (6202/53,150). After excluding patients without fibrinogen, it rises to be 15.7% (3071/19,512). As to AKI patients included in our analysis of fibrinogen level, the overall in-hospital mortality is 31.7% (1874/5914).

### Data extraction

Structured Query Language was used to extract data in the first 24 h of ICU admission, including patient gender, age, use of vasopressor, ventilator and renal replacement therapy (RRT), Simplified Acute Physiology Score (SAPS) II, Sequential Organ Failure Assessment (SOFA) scores, and laboratory tests. Laboratory tests include fibrinogen, ATIII, white blood cell (WBC), hemoglobin, albumin, platelet, potassium, sodium, lactate, total bilirubin, glucose, prothrombin time (PT), and partial thromboplastin time (PTT). Max creatine during ICU hospitalization was also acquired. Admission diagnoses including sepsis, acute myocardial infarction (AMI), heart failure (HF), cerebral infarction, cerebral hemorrhage, acute respiratory failure, and comorbidities include liver cirrhosis, hypertension, chronic obstructive pulmonary disease (COPD), chronic kidney disease (CKD), atrial fibrillation (AF), and thrombosis were also extracted. These extracted variables were used as independent variables. In addition to being primary endpoint, in-hospital mortality was also used as dependent variables together with survival time (LOS in ICU) in Cox proportional hazards regression.

### Statistical analysis

There are two cohort studies separately based on the levels of fibrinogen and ATIII. Patients with fibrinogen were stratified into three groups: (1) low level <150 mg/dL; (2) normal level 150–400 mg/dL; (3) high level >400 mg/dL. Patients with AT3 were stratified into two groups: (1) low level <80%; (2) normal level 80%–130%. We structured four multivariate models regarding fibrinogen groups. Initial model adjusts for none. Model 1 adjusts for: age, gender, cerebral infarction, cerebral hemorrhage, and thrombosis; Model 2 adjusts for model 1 plus AMI, HF, liver cirrhosis, hypertension, chronic obstructive pulmonary disease, CKD, atrial fibrillation, sepsis, and acute respiratory failure. Model 3 adjusts for model 2 plus WBC, hemoglobin, albumin, platelet, potassium, sodium, lactate, bilirubin, glucose, PT and PTT, creatine maximum, use of RRT, and ventilator. Model 4 adjusts for model 3 plus SOFA and SAPSII scores. As to ATIII, we only conducted univariate COX regression analysis owing to limited sample size.

Numerical variables were shown as mean ± standard deviation (SD) on the premise of normal distribution. Categorical variables were presented as frequency (percent). One-way ANOVA or students *t*-test was applied to normally distributed continuous variables to determine difference between groups. Kruskal–Wallis test was utilized in non-normally distributed continuous variables and Chi-square test was used in categorical data. Cox proportional hazards regression model was applied to calculate the hazard ratio (HR) with 95% confidence intervals (CIs) and estimate the relationship between coagulation parameters and in-hospital mortality. The normal level group was treated as the reference group. We choose covariates for multivariable analysis based on the following principles: (1) whether the covariates will make a difference to production and consumption of fibrinogen in humans; (2) whether the covariates, especially some diagnoses, will influence the severity of illness or even leading to in-hospital death directly; (3) the proportion of missing values should be less than 10%; (4) referring to similar published papers.

Subgroup analysis was conducted to assess association between fibrinogen and in-hospital mortality.

Interactions with factors utilized in model 3 for adjustment were also evaluated.

Nonlinear relationships between fibrinogen or ATIII activity and in-hospital mortality were assessed using restricted cubic spline (RCS) curves. Kaplan–Meier method was used to estimate cumulative incidence of mortality by fibrinogen levels or ATIII levels. Log-rank tests were conducted to identify the differences in survival distribution among three fibrinogen levels and two ATIII activity levels. Receiver-operating characteristic (ROC) curve was plotted and area under curve (AUC) was calculated to evaluate predictive ability of fibrinogen or ATIII plus SOFA score or SAPSII score on in-hospital mortality.

All statistical analyses were performed using SPSS statistics 22 software (SPSS Inc., IBM, USA), R V.3.6.3 (R Foundation for Statistical Computing, Vienna, Austria) and Stata 16.0 (StataCorp LLC, Texas, USA).

## Results

### Population characteristics

A total of 5914 eligible patients were enrolled in fibrinogen cohort study and 115 patients were eligible for ATIII cohort study. Characteristics of enrolled patients were shown in Table 1. In fibrinogen cohorts, 2250 women and 3664 men were included and they have a mean age of 65 ± 16 years. Patients with low level of fibrinogen tend to be younger with a history of liver cirrhosis and without a history of CKD, as well as higher value of lactate, bilirubin, PTT, sofa score, and mortality. There are 53 females and 62 males with a mean age of 50 ± 17 years in ATIII cohorts (Table 2).

**Table 1.** Characteristics of patients by fibrinogen level.

Characteristics	Fibrinogen (mg/dL)			*p* value
	Low level (<150)	Normal level (150–400)	High level (>400)	
*N*	791	3162	1961	–
Age, years	59.2 ± 15.8	65.4 ± 15.4	65.7 ± 15.4	<.001
Gender, *n* (%)				.029
Female	332 (42.4)	1175 (37.3)	743 (38.2)	–
ICU LOS, day	6.9 ± 8.4	6.0 ± 7.6	7.0 ± 9.0	<.001
Admission diagnoses, *n* (%)				
Sepsis	311 (37.4)	984 (30.7)	1060 (53.0)	<.001
AMI	41 (4.9)	351 (10.9)	185 (9.3)	<.001
Heart failure	155 (18.7)	1213 (37.9)	747 (37.4)	<.001
Cerebral infarction	29 (3.5)	152 (4.7)	111 (5.6)	.063
Cerebral hemorrhage	10 (1.2)	45 (1.4)	40 (2.0)	.16
Acute respiratory failure	395 (47.5)	1209 (37.8)	947 (47.4)	<.001
Comorbidities, *n* (%)				
Liver cirrhosis	399 (48.0)	506 (15.8)	81 (4.1)	<.001
Hypertension	149 (17.9)	601 (18.8)	422 (21.1)	.059
COPD	43 (5.2)	238 (7.4)	161 (8.1)	.026
CKD	171 (20.6)	989 (30.9)	663 (33.2)	<.001
Atrial fibrillation	205 (24.7)	1275 (39.8)	746 (37.3)	<.001
Thrombosis	126 (15.2)	370 (11.6)	270 (13.5)	.009
Laboratory examinations				
WBC, 10^9^/L	13.8 ± 25.9	13.5 ± 16.9	14.6 ± 16.1	.1
Creatine, mEq/L	3.5 ± 2.2	3.1 ± 2.3	3.4 ± 2.5	<.001
Hemoglobin, g/dL	10.6 ± 2.7	11.2 ± 2.6	11.0 ± 2.5	<.001
Albumin, g/dL	2.9 ± 0.8	3.2 ± 0.7	3.0 ± 0.7	<.001
Platelet, 10^9^/L	127.1 ± 79.3	190.9 ± 106.6	223.3 ± 133.0	<.001
Potassium, mEq/L	4.5 ± 1.0	4.4 ± 1.0	4.5 ± 1.0	.16
Sodium, mEq/L	135.5 ± 7.1	137.3 ± 6.0	137.3 ± 6.6	<.001
Lactate, mmol/L	4.5 ± 4.3	3.0 ± 2.8	2.7 ± 2.2	<.001
Bilirubin, mg/dL	6.8 ± 9.3	2.5 ± 5.7	1.4 ± 2.8	<.001
Glucose, mg/dL	142.5 ± 79.0	152.6 ± 100.0	163.2 ± 116.1	<.001
PT, s	23.5 ± 13.2	17.9 ± 12.0	19.1 ± 16.0	<.001
PTT, s	49.0 ± 27.8	38.7 ± 23.4	36.1 ± 19.8	<.001
Score				
SOFA	12.4 ± 4.7	9.1 ± 4.4	8.6 ± 4.4	<.001
SAPSII	49.4 ± 16.2	45.7 ± 15.3	45.9 ± 16.3	<.001
Treatment				
Vasopressor	569 (68.5)	2015 (62.9)	1130 (56.5)	<.001
Ventilator	747 (89.9)	2833 (88.5)	1754 (87.7)	.25
RRT	256 (30.8)	551 (17.2)	350 (17.5)	<.001
AKI stage, *n* (%)				<.001
Stage 1	164 (19.7)	956 (29.9)	649 (32.5)	–
Stage 2	181 (21.8)	971 (30.3)	520 (26.0)	–
Stage 3	486 (58.5)	1275 (39.8)	831 (41.6)	–
In hospital mortality, *n* (%)	394 (47.4)	887 (27.7)	593 (29.6)	<.001

AMI: acute myocardial infarction; COPD: chronic obstructive pulmonary disease; CKD: chronic kidney disease; WBC: white blood cell; PT: prothrombin time; PTT: partial thromboplastin time; SOFA: Sequential Organ Failure Assessment scores; SAPSII: Simplified Acute Physiology Score; RRT: renal replacement therapy.

**Table 2.** Characteristics of patients by ATIII level.

Characteristics	Antithrombin III (%)		*p* value
	Low level (<80)	Normal level (80–130)	
*N*	76	39	–
Age, years	50.5 ± 17.5	49.3 ± 15.7	.64
Gender, *n* (%)			.70
Female	36 (47.4)	17 (43.6)	–
ICU LOS, day	12 ± 13.5	6.6 ± 7.1	0.019
Admission diagnoses, *n* (%)			
Sepsis	37 (48.7)	2 (5.1)	<.001
AMI	11 (14.5)	0 (0)	.01
Heart failure	18 (23.7)	5 (12.8)	.17
Cerebral infarction	10 (13.2)	20 (51.3)	<.001
Cerebral hemorrhage	5 (6.6)	6 (15.4)	.13
Acute respiratory failure	43 (56.6)	12 (30.8)	.009
Comorbidities, *n* (%)			
Liver cirrhosis	7 (9.2)	1 (2.6)	.18
Hypertension	6 (7.9)	12 (30.8)	.001
COPD	3 (3.9)	2 (5.1)	.77
CKD	10 (13.2)	5 (12.8)	.96
Atrial fibrillation	18 (23.7)	2 (5.1)	.01
Thrombosis	32 (42.1)	8 (20.5)	.02
Laboratory examinations			
WBC, 10^9^/L	12.9 ± 6.7	11.8 ± 4.7	.32
Creatine, mEq/L	3.1 ± 2.7	2.3 ± 3.5	.15
Hemoglobin, g/dL	11.9 ± 2.5	12.9 ± 2.4	.99
Albumin, g/dL	3.1 ± 0.8	3.7 ± 0.8	.99
Platelet, 10^9^/L	193.4 ± 94.1	214.2 ± 78.4	.88
Potassium, mEq/L	4.4 ± 1.1	4.1 ± 0.7	.15
Sodium, mEq/L	136.1 ± 5.5	139.1 ± 3.6	.004
Lactate, mmol/L	3.0 ± 2.7	2.7 ± 2.0	.22
Bilirubin, mg/dL	1.4 ± 1.6	0.5 ± 0.3	.002
Glucose, mg/dL	160.9 ± 98.7	148.5 ± 89.5	.51
PT, s	16.9 ± 8.1	14.2 ± 6.1	.07
PTT, s	42.1 ± 29.7	30.6 ± 13.2	.024
Score			
SOFA	9.7 ± 5.2	5.2 ± 2.9	<.001
SAPSII	39.2 ± 14.8	29.1 ± 10.6	<.001
Treatment			
Vasopressor	50 (65.8)	10 (25.6)	<.001
Ventilator	70 (92.1)	28 (71.8)	.003
RRT	21 (27.6)	2 (5.6)	.004
AKI stage, *n* (%)			.007
Stage 1	12 (15.8)	12 (30.8)	–
Stage 2	28 (36.8)	20 (51.3)	–
Stage 3	36 (47.4)	7 (17.9)	–
In hospital mortality, *n* (%)	26.3	7.7	.02

AMI: acute myocardial infarction; COPD: chronic obstructive pulmonary disease; CKD: chronic kidney disease; WBC: white blood cell; PT: prothrombin time; PTT: partial thromboplastin time; SOFA: Sequential Organ Failure Assessment scores; SAPSII: Simplified Acute Physiology Score; RRT: renal replacement therapy.

### Fibrinogen and in-hospital mortality

**Figure 1.**
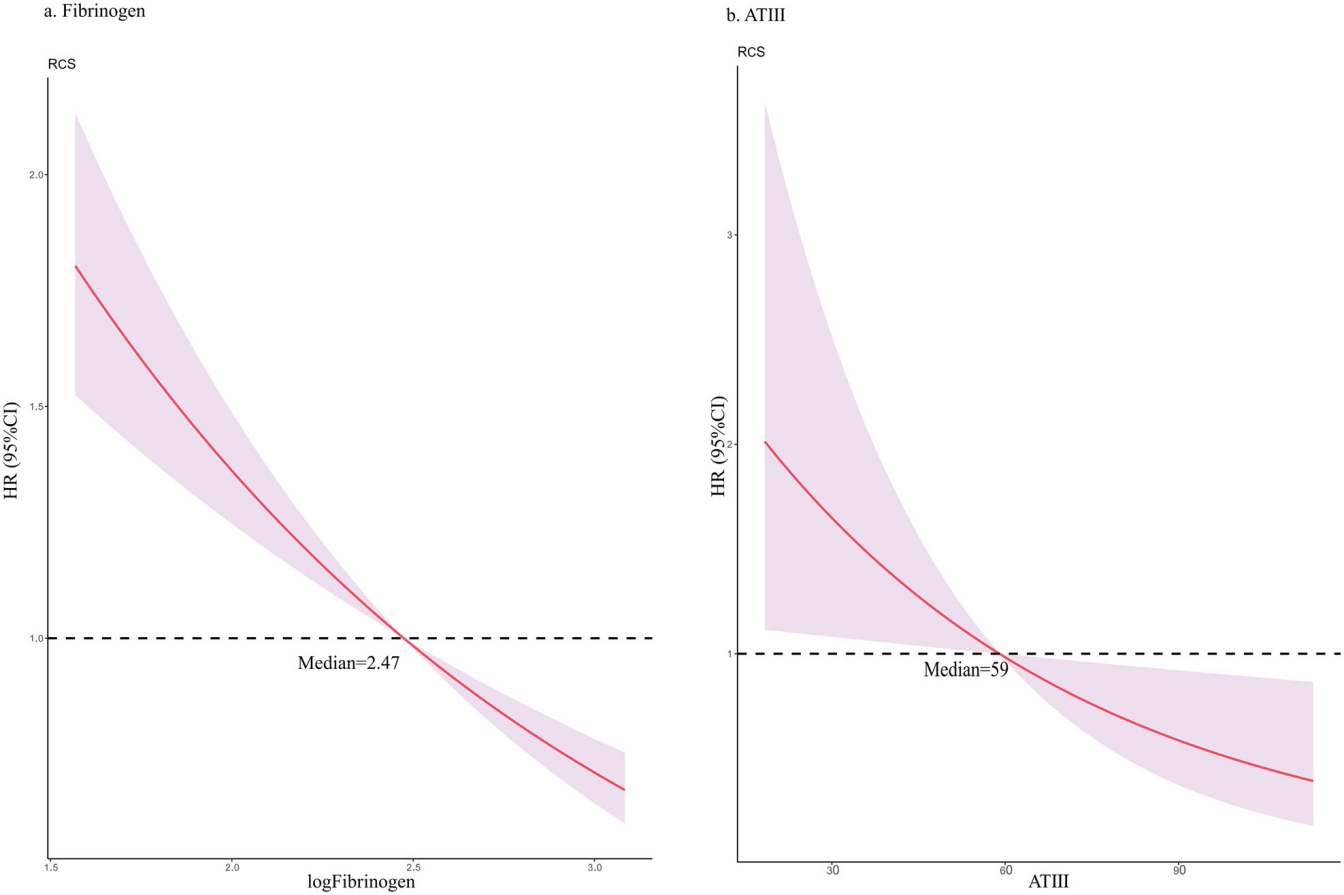
RCS for the association between (a) log transformed fibrinogen and in-hospital mortality, (b) ATIII and in-hospital mortality. The HR of median was used as baseline, respectively 2.47 for log transformed fibrinogen and 59 for ATIII.

**Figure 2.**
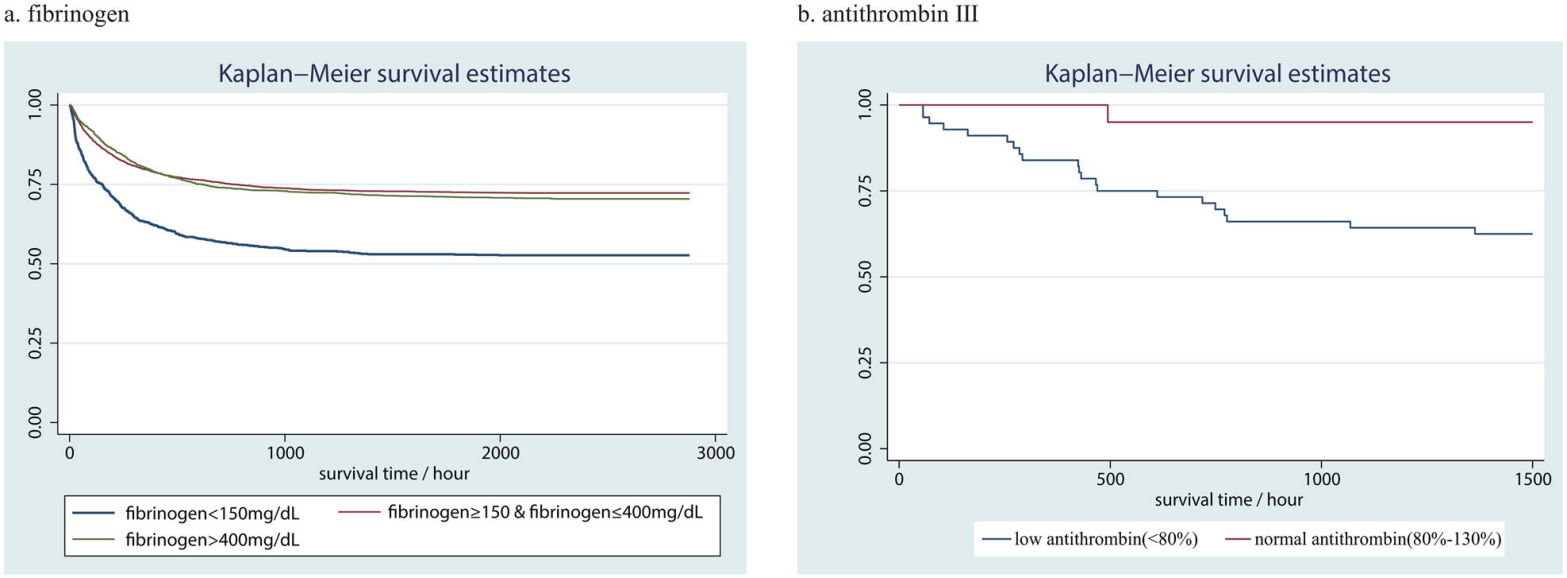
Kaplan–Meier survival curve for (a) fibrinogen and (b) ATIII. Log-rank test for fibrinogen *p* < .001 and ATIII *p* = .023.

The results of univariate and multivariate Cox regression concerning fibrinogen level were summarized in Table 3. The results of other variables adjusted for multivariate regression in model 4 can be seen in Supplementary Table 1. In the unadjusted model, the HR and 95% CIs (95% CIs) of low fibrinogen group and high fibrinogen group were 2.01 (1.79, 2.27) and 1.06 (0.95, 1.17) separately. In model 4, the HR (95% CIs) turned to be 1.29 (1.13, 1.48) and 0.91 (0.81, 1.01). The RCS curve based on model 3 demonstrated nearly linear curve relationship between log-transformed fibrinogen and in-hospital mortality (Figure 1a). Kaplan–Meier survival curve showed significantly lower survival rate in low fibrinogen level group compared with normal or high-level group (Log-rank test *p* < 0.001) (Figure 2a).

**Table 3.** Relationship between fibrinogen and in-hospital mortality.

	Low level (<150 mg/dL)		High level (>400 mg/dL)	
	HR (95% CI)	*p* value	HR (95% CI)	*p* value
Unadjusted	2.01 (1.79, 2.27)	<.001	1.06 (0.95, 1.17)	.29
Model 1	2.11 (1.86, 2.37)	<.001	1.05 (0.94, 1.16)	.41
Model 2	1.75 (1.54, 1.99)	<.001	0.85 (0.76, 0.95)	.005
Model 3	1.36 (1.19, 1.56)	<.001	0.86 (0.77, 0.96)	.007
Model 4	1.29 (1.13, 1.48)	<.001	0.91 (0.81, 1.01)	.083

Reference group was normal fibrinogen group (150–400 mg/dL).

Model 1 adjusted for age, gender, cerebral infarction, cerebral hemorrhage, and thrombosis.

Model 2 adjusted for covariates in model 1 + AMI, heart failure, liver cirrhosis, hypertension, chronic obstructive pulmonary disease, chronic kidney disease, atrial fibrillation, sepsis, and acute respiratory failure.

Model 3 adjust for covariates in model 2 + WBC, hemoglobin, albumin, platelet, potassium, sodium, lactate, bilirubin, glucose, prothrombin time and partial thromboplastin time, creatine max, use of RRT, and ventilator.

Model 4 adjust for covariates in model 3 + SOFA and SAPSII score.

### ATIII and in-hospital mortality

The HR (95% CI) of low ATIII level versus normal level was 3.73 (1.11, 12.54). The RCS curve demonstrated small-radian curve relationship between ATIII and in-hospital mortality (Figure 1b). Kaplan–Meier survival curve also showed significantly lower survival rate in low-level group compared with normal level group (Log-rank test *p* = .023) (Figure 2b).

### Subgroup analysis

Most sub-populations showed similar hazards ratio concerning the association between fibrinogen and in-hospital mortality. Sepsis, atrial fibrillation, acute respiratory failure, the use of renal replacement therapy, hemoglobin, lactate, PT, and PTT interacted significantly with fibrinogen in adjusted model 3 (Table 4). In AKI stage 3 patients, fibrinogen seems less effective in predicting in-hospital death than AKI stage 2 or stage 1 patients (AKI stage 3, HR 95% CI 1.15 [0.98, 1.36] *p* = .088; AKI stage, 2.18 [1.58, 3.22] *p* < .001; AKI stage 1, 2.05 [1.47, 2.85] *p* < .001).

**Table 4.** Subgroup analysis of the association between fibrinogen and in hospital mortality based on Model 3.

Characteristics	*N*	HR (95% CI)	*p* value	*p* for interaction
Sepsis				<.001
No	3603	1.77 (1.45, 2.16)	<.001	–
Yes	2311	1.23 (1.02, 1.47)	.028	–
CKD				.83
No	4135	1.32 (1.13, 1.53)	<.001	–
Yes	1779	1.67 (1.24, 2.24)	.001	–
Atrial fibrillation				.002
No	3753	1.44 (1.23, 1.69)	.001	–
Yes	2161	1.19 (0.92, 1.54)	.19	–
Acute respiratory failure				.017
No	3428	1.68 (1.37, 2.07)	<.001	–
Yes	2486	1.19 (1.00, 1.41)	.055	–
Lactate, mmol/L				.005
<2	2752	1.70 (1.30, 2.23)	<.001	–
≥2	3162	1.25 (1.07, 1.46)	.004	–
Bilirubin, mg/dL				<.001
<1.5	4116	1.47 (1.18, 1.82)	<.001	–
≥1.5	1798	1.24 (1.04, 1.48)	.015	–
RRT				.016
No	4779	1.44 (1.22, 1.71)	<.001	–
Yes	1135	1.24 (0.99, 1.55)	.058	–
AKI stage				<.001
Stage 1	1735	2.05 (1.47, 2.85)	<.001	–
Stage 2	1635	2.18 (1.58, 3.22)	<.001	–
Stage 3	2544	1.15 (0.98, 1.36)	.088	–
WBC, 10^9^/L				<.001
4–11	2685	1.42 (1.14, 1.77)	.001	–
<4	358	1.36 (0.78, 2.38)	.282	–
>11	2871	1.40 (1.17, 1.69)	<.001	–
Creatine, mEq/L				.53
<1.2	179	2.28 (0.67, 7.76)	.19	–
≥1.2	5735	1.39 (1.21, 1.59)	<.001	–
Hemoglobin, g/dL				.014
<12	3746	1.36 (1.16, 1.60)	<.001	–
≥12	2135	1.38 (1.08, 1.75)	.01	–

### Receiver operating characteristic curve

**Figure 3.**
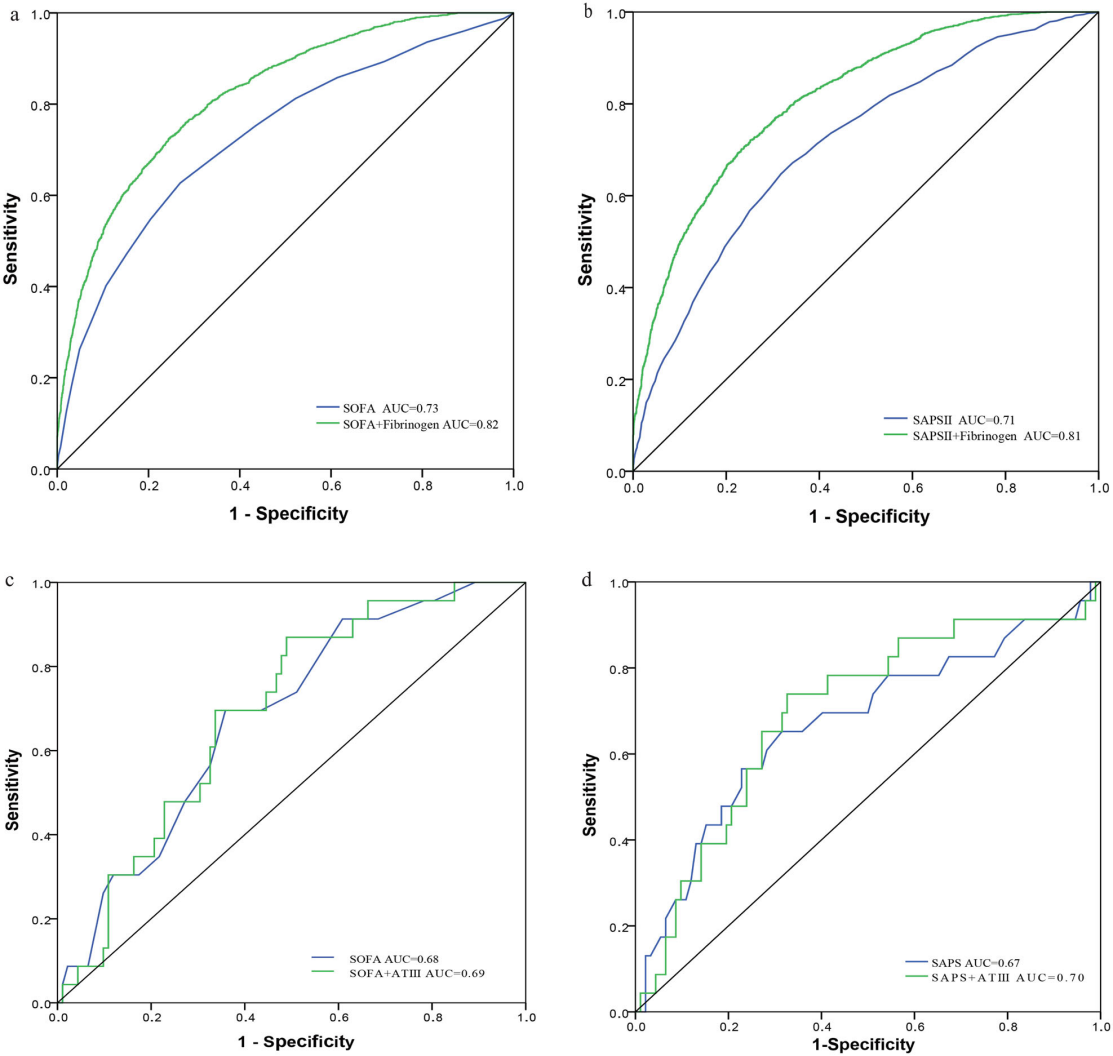
ROC curves for the prediction of in-hospital mortality. (a) The ability of SOFA scores versus fibrinogen plus SOFA scores to predict in-hospital mortality, *p* = 0.027. (b) The ability of SAPSII scores versus fibrinogen plus SAPSII scores, *p* = .038. (c) The ability of SAPSII scores versus ATIII plus SOFA scores, *p* = 0.26. (d) The ability of SAPSII scores versus ATIII plus SAPSII scores, *p* = .12.

The AUC for fibrinogen plus SOFA score was 0.82, rising from 0.73 of SOFA score alone (*p* = .027) (Figure 3a). Correspondingly, the AUC for SAPSII score plus fibrinogen and SAPSII alone were 0.81 and 0.71 respectively (*p* = .038) (Figure 3b).

However, in ATIII cohort study, the AUC for ATIII plus SOFA score was 0.69, just a little more capable of predicting than 0.68 of SOFA score alone (*p* = .26) (Figure 3c). Similarly, the AUC for ATIII plus SAPSII score and SAPSII alone were 0.70 and 0.67 respectively (*p* = .12) (Figure 3d).

## Discussion

In this study, we reported that coagulation parameters, especially fibrinogen and ATIII, are associated with all-cause in-hospital mortality among critically ill patients with AKI. To be more specific, patients with decreased fibrinogen or ATIII suffered from an increased risk of mortality, which appeared nearly linear relationship as you can see in RCS. Furthermore, the subgroup analysis demonstrated consistence of prognostic value in various sub-populations, despite the interactions of many covariates with fibrinogen, including sepsis, atrial fibrillation, acute respiratory failure, the use of renal replacement therapy, hemoglobin, lactate, PT and PTT.

Fibrinogen is a dimer composed of three pairs of disulfide-linked polypeptide chains (Aα, Bβ, and γ) [26] and synthesized by liver and endothelial cells of intestine [27], lung [28] and renal tubular [29]. In the term of biological mechanism, fibrinogen played a cross-talk role between coagulation and inflammation [18]. When inflammation happens, the conversion of fibrinogen into fibrin will be simultaneous, triggered by thrombin, a proteinase which can be inhibited by antithrombin [30]. Fibrin(ogen) was proved to facilitate leukocyte transmigration out of vessels and evoke leukocyte effector functions [30,31]. As to acute ischemic kidney injury, fibrinogen was reported to exert heterogeneous effects. A serious deficiency in fibrinogen causes significantly less survival and worse renal function, but partial reduction of fibrinogen could improve renal function and overall survival in mice [29]. Krishnamoorthy et al. [20] reported that mRNA expression of fibrinogen chains increased in the kidney after I/R injury and elevated urinary fibrinogen could serve as biomarker for AKI. Moreover, kidney fibrosis induced by folic acid could be inhibited by depletion of fibrinogen [32]. Some retrospective studies also claimed that elevated fibrinogen is associated with higher risk of AKI among patients who underwent percutaneous coronary intervention [33] and cardiac valve replacement [34]. Logically, fibrinogen seems a harmful substance in the development and prognosis of AKI. However, a propensity score-matching analysis revealed that patients with low fibrinogen are more likely to suffer from AKI compared with patients with normal fibrinogen who have underwent liver transplantation [19]. Meanwhile, fibrinogen deficiency was associated with higher mortality in kidney I/R rats [29]. Contradictory results emerged. So, it remains controversial as to the role of fibrinogen in AKI.

Here, we first report that low fibrinogen is a risk factor for mortality in critically ill patients with AKI. At the same time, we also noticed that AKI patients (*N* = 5914) tend to have higher serum fibrinogen than non-AKI patients (*N* = 13,479) (Supplementary Figure 1). A plausible explanation according to existing evidence is that fibrinogen will increase as a response to kidney injury such as ischemia reperfusion injury, like kim-1, which may be either protective or detrimental. In either case, the exhaustion of fibrinogen indicated the failure of source tissues like hepatocytes and renal tubular cells, thus predicting death of patients. But it’s mere speculation and requires further basic research.

Patients with liver cirrhosis were reported to be prone to AKI and further progress to hepatorenal syndrome causing high mortality [35]. The rate of liver cirrhosis was much higher in patients with low fibrinogen than patients with normal fibrinogen or high fibrinogen in our study. It seems that higher mortality in low fibrinogen patients could partly attribute to higher rate of liver cirrhosis. However, subgroup analysis showed that low fibrinogen remains risk factor despite the absence of cirrhosis (*p* for interaction = 0.63). To further exclude the interference of hepatocytes, our multivariate Cox analysis also includes bilirubin as a correction factor, which makes no difference to prognostic value of fibrinogen. Bilirubin interacted significantly with fibrinogen in Cox regression, which may be because both of them are synthesized by liver. But the subgroup analysis also revealed that low fibrinogen is always a risk factor for mortality in AKI patients despite the level of bilirubin.

Sepsis was proved to be both contributing factor and consequence of AKI [36,37], which could account for the interaction between sepsis and fibrinogen level. Sepsis associated acute kidney injury (S-AKI) will increase the risk of long-term comorbidities and cause exceedingly high mortality [4,38,39]. Correspondingly, we also found that sepsis was a risk factor for in-hospital mortality in univariate Cox regression (HR 2.39, 95% CI [2.19, 2.63], *p* < .001) and multivariate analysis (HR 1.63, 95% CI [1.45, 1.81], *p* < .001, model 4) in Supplementary Table 1. Moreover, fibrinogen is still capable of predicting in-hospital death in both sepsis (1.77 [1.45, 2.16], *p* < .001) and non-sepsis patients (1.23 [1.02, 1.47], *p* = .028) on the premise of AKI, as you can see in subgroup analysis.

In AKI stage 3 patients, fibrinogen seems less effective in predicting in-hospital death than AKI stage 2 or stage 1 patients. Actually, increased AKI severity was associated with increased mortality in critically ill patients [4], which is also proved in our study as you can see in Supplementary Table 1. The in-hospital mortality of AKI stage 3 patients in our cohort reaches 44.73% (1138/2544) while 21.65% (354/1635) in AKI stage 2 and 19.60% (340/1735) in AKI stage 1. The increase in mortality together with severity of AKI may contribute to the decreased efficiency of fibrinogen in predicting in-hospital death because deaths in critically ill AKI stage 3 patients are too common to be precisely forecasted, nearly half of them will die. Sometimes, in clinical practice, to predict the fate of a patient with nearly 50% probability of death is unnecessary because physicians will always pay more attention to such a person in great danger. Instead, in patients with moderate likelihood of death, a biomarker like fibrinogen which hints mortality will be helpful because it reminds physicians to be more careful and take more aggressive measures. In one word, fibrinogen is more effective in predicting in-hospital death in AKI stage 1 and stage 2 patients. Meanwhile, we also conducted univariate Cox regression analysis on critically ill patients without AKI. HR with CIs of low level of fibrinogen is 1.11 [0.92, 1.33] and *p* value is .27, which hints that the predictive value of low level of fibrinogen is unique to AKI patients and not very applicable to non-AKI patients in spite of similar trend.

The improvement or deterioration of AKI could contribute to survival or death of patients. Unfortunately, we failed to assess the prognosis of renal function for the lack of periodic reexamination of serum creatine, which means that some deaths may not be attributed to AKI. Some patients may have recovered from kidney injury, but finally died of other illness, making it less explicit and persuasive about the causality between AKI and in-hospital death.

As to ATIII, the limited sample size makes it difficult to further verify the authenticity of prognostic value through multivariate analysis. ATIII is a small glycoprotein produced by the liver, which will inactivate thrombin in plasma, thus inhibiting coagulation cascade and playing a crucial role in anticoagulation *in vivo* [40]. Emerging evidence proved that ATIII exerts anti-inflammatory [41–43] and renoprotective effects [21,22,44,45]. It has been proved that endogenous ATIII deficiency will worsen AKI [21] and exogenous administration will ameliorate kidney injury [22,45] and transition to chronic kidney disease [44]. It may have been enough to explain why low ATIII activity could predict in-hospital death based these studies. Moreover, patients with relatively low level of ATIII have higher probability of suffering from AKI when they underwent cardiac surgery, including valve replacement and coronary bypass grafting[21], so are patients undergoing coronary arteriography [22]. Simultaneously, low level of ATIII was identified as a risk factor for incidence of AKI in preeclampsia patients undergoing cesarean section [24] and patients undergoing liver transplantation [23] through logistic regression analyses. Given all previous studies, low level of ATIII is significantly associated with higher incidence of AKI induced by either ischemic reperfusion or contrast. Our study further showed that low level of ATIII may also be associated with the prognosis of AKI patients.

There are several limitations in our study. First, we used value of fibrinogen or ATIII activity upon admission to ICU and ignore the exogenous administration of them during ICU stay, which may exert positive or negative effects. Second, the data in our cohort study come from a single center database. Though inclusion standards were set, selection bias was unavoidable. MIMIC-IV v1.0 removed data of follow-up. So, merely in-hospital mortality was analyzed, which may not reflect the prognosis of AKI comprehensively. Moreover, the prognosis of renal function was not evaluated, making the causality between AKI and in-hospital death less convincing. Finally, some inflammation-related parameters, like C-reactive protein (CRP), procalcitonin (PCT), and interleuin-6 (IL-6), are not included in our multivariate analysis due the excessive proportion of missing data.

## Conclusions

Low fibrinogen is an independent predictor of in-hospital mortality in critically ill patients with AKI, especially stage 1 and stage 2. Low ATIII activity is also likely to impact the risk of in-hospital death.

## Supplementary Material

Supplemental Material

## Data Availability

Publicly available datasets were used in this study. These can be found in MIMIC-IV at https://doi.org/10.13026/s6n6-xd98.
