# Supplementary figures and images for: Fibrinogen and antithrombin III are associated with in-hospital mortality among critically ill patients with acute kidney injury
Source: Ren Fail. 2022 Nov 10;44(1):1948–57. doi: 10.1080/0886022X.2022.2142138 (PMC9662049; doi:10.1080/0886022X.2022.2142138)

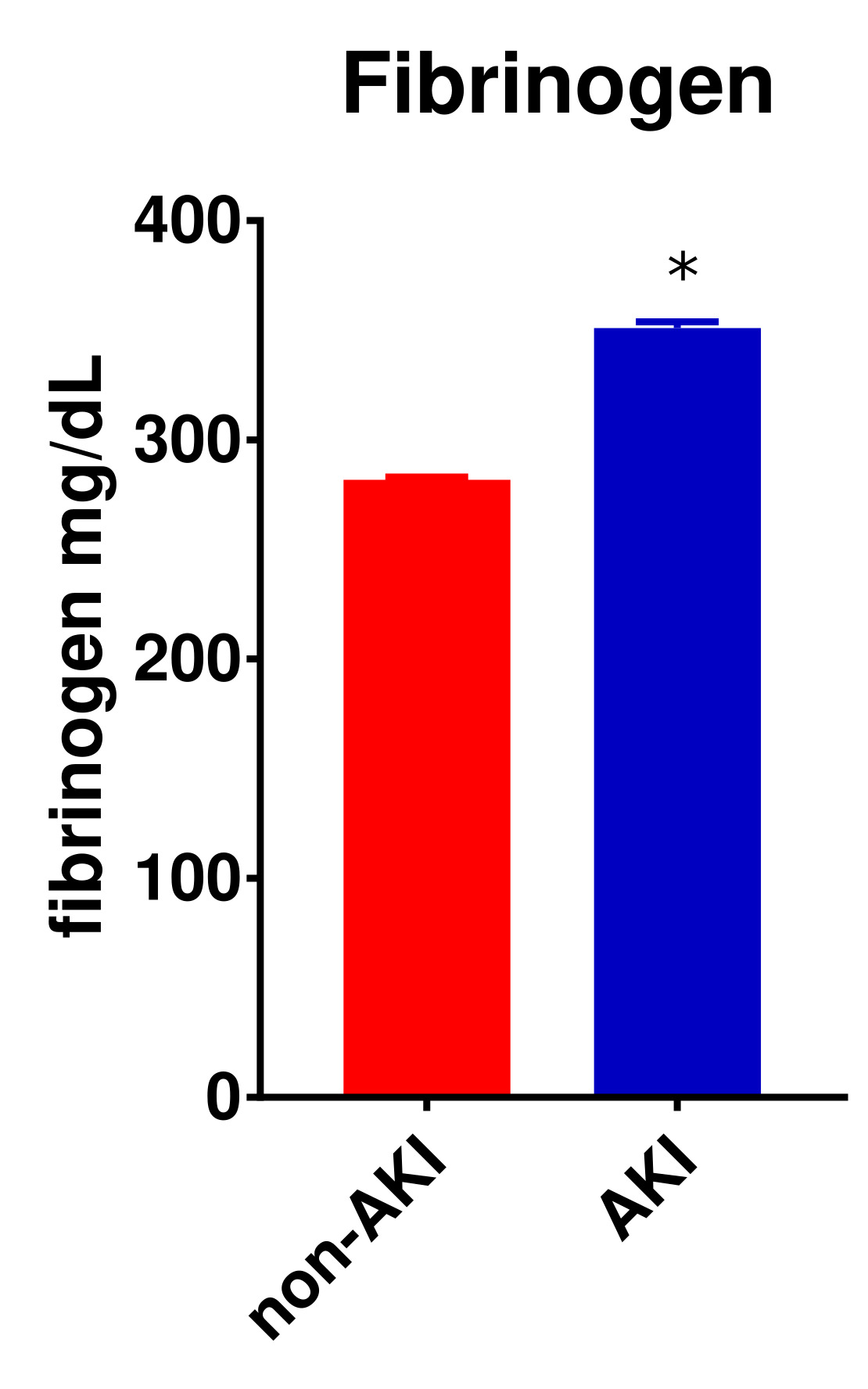

Supplement: Supplemental Material [file IRNF_A_2142138_SM9831.jpg]
